# Dynamic Fumonisin B_2_ Production by *Aspergillus niger* Intented Used in Food Industry in China

**DOI:** 10.3390/toxins9070217

**Published:** 2017-07-09

**Authors:** Xiaomin Han, Hongru Jiang, Jin Xu, Jing Zhang, Fengqin Li

**Affiliations:** 1 Key Laboratory of Food Safety Risk Assessment, Ministry of Health, China National Center for Food Safety Risk Assessment, Beijing 100021, China; hanxiaomin@cfsa.net.cn (X.H.); xujin@cfsa.net.cn (J.X.); zhangjing@cfsa.net.cn (J.Z.); 2 National Institute for Nutrition and Health, Chinese Centre for Disease Control and Prevention, Beijing 100050, China; jianghr@ninh.chinacdc.cn

**Keywords:** *Aspergillus niger*, Fumonisin B_2_, Food industry, China

## Abstract

There are a total of 30 strains including 27 strains of *Aspergillus niger* intended used in Chinese food industry, two strains used as control and one strain isolated from corn for fumonisin (FB) production on 3 media. It was found that FB_2_ production by *A. niger* was function-dependent and highly related to culture media, as well as incubation time. All strains studied were unable to produce FB_1_ and FB_3_. Almost all strains were found to produce FB_2_ on corn, rice and wheat bran. Based on their intended use in the food industry, the higher level of FB_2_ producers were strains used for saccharifying enzyme (*n* = 13) production, followed by organic acid (*n* = 6), tannase (*n* = 7) and β-galactosidase (*n* = 1) production, with the FB_2_ mean level of 3553–10,270 μg/kg, 1059–12,036 μg/kg, 3–7 μg/kg and 2–4 μg/kg on corn, 5455–9241 μg/kg, 559–2190 μg/kg, 4–9 μg/kg and 6–10 μg/kg on rice, 5959–7709 μg/kg, 9491–17,339 μg/kg, 8–14 μg/kg and 120–222 μg/kg on wheat bran, respectively. Comparatively, strains of *Fusarium verticillioide* were capable of producing fumonins simultaneously with broader spectrum including FB_1_, FB_2_ and FB_3_, but at a much lower level. In conclusion, it is necessary to evaluate FB_2_ production by *A. niger* before intended use in the food processing industry.

## 1. Introduction

Fumonisins were firstly isolated from the *Fusarium verticillioides* (formerly *F. moniliforme*) strain MRC 826 by Gelderblom et al in 1984 [1]. These toxins are divided into four series: A, B, C and P, based on their structures, with FB_1_, FB_2_ and FB_3_ being the most abundant naturally occurring homologues in foods [2,3]. Since FB_1_ and FB_2_ have been shown to be potent cancer promoters and possible carcinogens, consumption of foods and feeds contaminated by fumonisins has been considered in relation to the high incidence of human esophageal cancer in some areas of China and South Africa [4,5,6]. Therefore, B type fumonisins have become a new research hotspot after aflatoxins.

*F.verticillioides* and *F. proliferatum* are the main fumonisin producing fungi worldwide [7]. In the past 20 years, most studies on fumonisin production and the corresponding toxigenic genes are all focusing on these two *Fusarium* species [8]. However, recent reports have confirmed the FB_2_ producing ability of *A. niger*. About 77%, 77%, 50%, 76% and 72% strains of *A. niger* isolated from grape, grape raisin, grape mash, coffee and maize kernels, respectively, are capable of producing FB_2_ [9,10,11,12,13]. Besides, 42% strains of *Aspergillus section Nigri* isolated from corn also shown the capability of FB_2_ production [14]. *A. niger* is widely used in production of some foods and food additives and it has been granted as a “Generally Regarded As Safe” by US Food and Drug Administration in 1987. However, FB_2_ was detected for the first time in *A. niger* fermented products by Frisvad et al. and the safety of *A. niger* has drawn people’s attention thereafter [15]. According to Frisvad et al., about 83% strains of *A. niger* including NRRL337, NRRL3112 and NRRL3122 used for production of organic acids, extracellular enzymes, etc. could produce FB_2_ [16]. But studies on FB_2_ production by strains of *A. niger* used in Chinese food industry has not been reported so far. The objective of this study was to evaluate FB_2_ production on different media by *A. niger* intended used for Chinese food industry on different experimental conditions, to elucidate the differences in fumonisin production pattern between *A. niger* and *F. verticillioides*.

## 2. Results

### 2.1. Time Course of FB_2_ Production by A. niger

Totally, 30 strains of *A. niger* including 27 strains intended used for Chinese food industry, two types of strains from the American Type Culture Collection, and one strain isolated from corn were employed to survey FB_1_, FB_2_ and FB_3_ production. Among them, 27 strains of *A. niger* intended used for food industry were classified into four categories based on their functions: saccharifying enzyme, organic acid, tannase and β-galactosidase producers. All strains were unable to produce FB_1_ or FB_3_, which was consistent with the previous report [16,17,18,19,20]. Almost all strains yield FB_2_ on rice, corn and wheat bran at different time intervals of day 7, day 14, day 21, and day 28, respectively. Besides, the variation of FB_2_ production by *A. niger* was considerably different for strains with different functions in Chinese food processing industry (Table 1). Particularly, significant differences in FB_2_ production among strains with same function in the food industry were also observed (Table 1). 

Thirteen strains of *A. niger* used for saccharifying enzyme production as well as six strains for organic acid production were all positive for FB_2_ at high levels at all time intervals on three media (Table 1). It should be pointed out that only one strain used for organic acid production coded OA-06 could produce FB_2_ at much lower levels on day 21 on corn (123 μg/kg) and rice (5 μg/kg) as well as on day 28 on wheat bran (2 μg/kg), respectively. Comparatively, saccharifying enzyme producers yielded FB_2_ at considerablely higher levels than those of organic acid producers on day 7 and day 14 on both corn and rice. While the organic acid producers were positive for FB_2_ at a higher average level than the saccharifying enzyme producers on wheat bran at all time intervals (Table 1).

A total of 1 out of 7 *A. niger* strains intended used for tannase production produced FB_2_ on three media with concentrations less than 30 μg/kg. Two strains coded TA-03 and TA-07 were negative for FB_2_ on rice and wheat bran at any time interval but positive on corn. The remainder of the four strains yielded FB_2_ occasionally on one or two media with the maximum concentration lower than 8 μg/kg at all of the time intervals. One strain of *A. niger* coded GA-01 intended used for beta-galactosidase production produced FB_2_ with levels lower than 4 μg/kg and 10 μg/kg on corn and rice, respectively, but a bit higher level on wheat bran (maximum: 138 μg/kg) was found. It is worth pointing out that FB_2_ production is strain function-dependent: strains intended used for saccharifying enzyme production are the highest FB_2_ producers followed by the organic acid, tannase and beta-galactosidase producers. It can be concluded that *A. niger* could produce higher levels of FB_2_ as the culture time increased for some strains.

### 2.2. Effect of Media on Dynamic Production of FB_2_ for Strains of A. niger Intended Used in Different Functions

FB_2_ production profile of *A. niger* varied in their function (Table 2). In the case of 13 saccharifying enzyme producers, the average FB_2_ level on wheat bran increased steadily with the maximum of 7709 μg/kg on day 28. Whereas the average FB_2_ concentration on rice increased significantly in the first seven days, and then stabilized until day 14, followed by increasing and reaching the maximum of 9241 μg/kg on day 28. However, the average FB_2_ concentration on corn increased during the first 14 days with a maximum of 10,270 μg/kg on day 14, and thereafter decreased on day 21 and day 28. Hence, much higher average concentration of FB_2_ on corn was observed in comparison with those on rice and wheat bran for some intervals. 

Regarding the organic acid producers, the average concentration of FB_2_ on corn increased gradually and reached a maximum average of 12,306 μg/kg on day 28. The same FB_2_ production profile was observed on rice but with an average FB_2_ level much lower than those on corn. However, much higher FB_2_ with the maximum average of 17,339 μg/kg on day seven was detected on wheat bran in comparison with those either on corn or on rice. This observation, therefore, indicated that wheat bran available to *A. niger* growth resulted in an increased concentration of FB_2_. Comparatively, lower levels of FB_2_ were synthesized by seven strains and one strain of *A. niger* intended used for tannase and β-galactosidase production, with the maximum level of 30 μg/kg and 222 μg/kg, respectively. Overall, wheat bran showed the highest average production of FB_2_ by tannase and β-galactosidase producers, with 2 to 33.5 times and 1.1 to 12.8 times higher than those on corn and rice, respectively. 

### 2.3. Distribution of FB_2_ Produced by A. niger Intended Used in Chinese Food Industry

**Figure 1:**
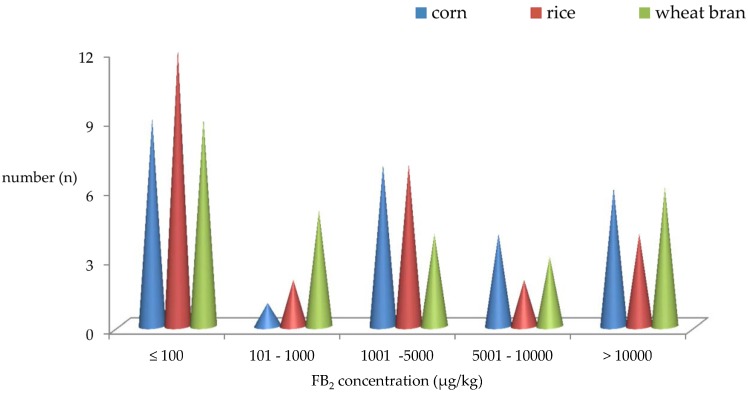
Distribution of FB_2_ Concentrations Produced by 27 Strains of *A. niger* Intended Used in Chinese Food Industry. *n* = FB_2_ positive strain number for *A. niger* intended used in Chinese food industry.

Distributions of the average FB_2_ concentration produced by 27 strains of *A. niger* at different time intervals on 3 media are given in Figure 1. All 27 strains of *A. niger* tested were divided into five groups: ≤100 µg/kg, 101–1000 µg/kg, 1001–5000 µg/kg, 5001–10,000 µg/kg and >10,000 µg/kg based on their FB_2_ average levels at all of the time intervals on different mediums. Two strains TA-03 and TA-07 were free from detectable amounts of FB_2_ on rice and wheat bran at any time intervals. Six (6/27, 22%), 4 (4/27, 15%) and 6 (6/27, 22%) strains produced FB_2_ at an average level higher than 10000 µg/kg, with a maximum of 70488 μg/kg, 54284 μg/kg and 38094 μg/kg on corn, rice and wheat bran, respectively. While, 4 (4/27, 15%), 2 (2/27, 7%) and 3 (3/27, 11%) strains of *A. niger* synthesized FB_2_ at the average concentration between 5001 µg/kg and 10000 µg/kg on rice, corn and wheat bran, respectively. However, 7 (7/27, 26%), 7 (7/27, 26%) and 4 (4/27, 15%) strains yielded FB_2_ ranged from 1001 µg/kg to 5000 µg/kg on corn, rice, and wheat bran, respectively. Only the one (1/27, 4%), two (2/27, 7%) and five (5/27, 19%) strains yielded FB_2_ ranging from 101 µg/kg to 1000 µg/kg on corn, rice and wheat bran, respectively. It is worth pointing out that one third strains can synthesize FB_2_ at an average level below 100 µg/kg on three media. Therefore, there is a trend that more FB_2_ were produced on wheat bran and corn followed by rice. As mentioned above, screening of toxigenicity for strains of *A. niger* on different mediums should indeed be a priority before use in food production in order to keep the target food or food additives free from FB_2_.

One type strain of *A. niger* coded ATCC30557 produced FB_2_ at the highest level of 5764 μg/kg on rice, 2194 μg/kg on corn and 16 μg/kg on wheat bran, respectively on day 14. Whereas another type strain of *A. niger* coded ATCC16404 used as a reference in antifungal activity assay yielded FB_2_ at the level less than 40 μg/kg at all time intervals on rice and corn, no FB_2_ was detected on wheat bran other than 2 μg/kg on day 28. It should be emphasized that a strain isolated from corn synthesized FB_2_ and reached the highest of 23,700 μg/kg and 13,342 μg/kg on rice and corn on day 28, respectively, but the highest level of 8718 μg/kg was found on wheat bran on day 21. This revealed that wheat, corn and rice will be potentially contaminated with FB_2_ during the storage once they are invaded by *A. niger*.

### 2.4. Comparison of Fumonision Production Produced by A. niger and F. verticilliodes

**Figure 2:**
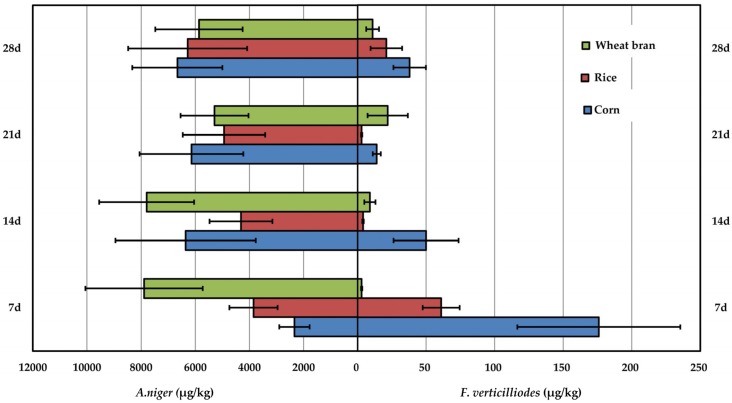
FB_2_ Average Concentrations for *A. niger* and *F. verticilliodes* (µg/kg).

The average FB_2_ production by both 30 strains of *A. niger* and 31 strains of *F. verticilliodes* are shown in Figure 2. It was found that the average FB_2_ levels yielded by *A. niger* on three media at all time intervals from day 7 to day 28 were in the range from 2339 μg/kg to 6661 μg/kg for corn, 3851 μg/kg and 6283 μg/kg for rice, 5291 μg/kg and 7891 μg/kg for wheat bran, respectively, much higher than those produced by strains of *F. verticilliodes* (in the range from 14 μg/kg to 176 μg/kg for corn, from 3 μg/kg to 61 μg/kg for rice, and from 3 μg/kg to 22 μg/kg for wheat bran, respectively). 

The average FB_2_ production by strains of *A. niger* on corn increased from 2339 μg/kg on day 7 to 6359 μg/kg on day 14, and deceased a little to 6141 μg/kg on day 21 and reached the maximum of 6661 μg/kg on day 28. In terms of rice, the average FB_2_ production increased gradually from 3581 μg/kg on day 7 to 4313 μg/kg on day 14, 4941 μg/kg on day 21, and reached the highest level of 6283 μg/kg on day 28. While the average production of FB_2_ on wheat bran was different with those on corn and rice, it reached the highest level of 7891 μg/kg on day 7, decreased to 7797 μg/kg on day 14, and down to the minimum level of 5291 μg/kg on day 21, respectively. On the other hand, the profile of average FB_2_ production by *F. verticilliodes* on corn and rice was similar to those by *A. niger*, reached the maximum of 176 μg/kg on corn and 61 μg/kg on rice on day 7, respectively and decreased gradually thereafter. Whereas, the production increased gradually from day 7 to day 21 on wheat bran, and reached the maximum of 22 μg/kg on day 21 followed by decreasing to 11 μg/kg on day 28. FB_2_ levels produced by *F. verticilloides* on corn and rice was different from those reported by Alberts et al [17], owing to the difference in both strains tested and the ingredients of the media employed.

Comparatively, it was found that the average FB_2_ production by *A. niger* was much higher than those by *F. verticilliodes* on any of 3 media (Figure 2), from 12 to 438 times higher for corn, 62 to 1646 times higher for rice and 239 to 2629 times higher for wheat bran, respectively. Therefore, it can be inferred that FB_2_ producing ability was fungi species-dependent. Additionally, media was one of the most important factors which influence FB_2_ production by fungi. Corn was a suitable medium for FB_2_ production by *F. verticilliodes* on almost all time intervals. Wheat bran supported the highest average level of FB_2_ production by *A. niger* on day 7 and day 14. Both *F. verticilliodes* and *A. niger* yielded the lowest level of FB_2_ on rice at any time intervals during the toxin production.

In addition, it is worth noting that the strains of *F. verticilliodes* could produce FB_1_, FB_2_ and FB_3_ simultaneously and the average FB_1_ and FB_3_ production by 31 strains of *F. verticilliodes* from day 7 to day 28 was 30 µg/kg to 166 µg/kg and 15 µg/kg to 164 µg/kg on corn, 8 µg/kg to 103 µg/kg and 5 µg/kg to 229 µg/kg on rice, and 7 µg/kg to 41 µg/kg and 2 µg/kg to 79 µg/kg on wheat bran, respectively. No FB_1_ and FB_3_ were synthesized by *A. niger* on any media at any time interval. Therefore, *A. niger* is a FB_2_ producer in comparison with *F. verticilliodes* that have a wider spectrum of fumonisins production.

## 3. Discussion

To the best of our knowledge, this is the first report on FB_2_ production by *A. niger* intended used for Chinese food production. *A. niger* is one of the most important industrial filamentous fungus extensively used for extracellular enzymes and organic acid production, biotransformation of xenobiotics [16,18], etc. However, concerns about the safety of this fungus have been raised with the discovery that some isolates can produce fumonisins on semi-synthetic medium such as rice corn steep agar (RC), Czapek yeast autolysate agar (CYA), CYA with 5% NaCl (CYAS), dichloran 18% glycerol agar (DG18), potato dextrose agar (PDA) and malt extract agar with Bacto malt extract (MEA), respectively [13,14,15,16,19,20,21,22]. Our findings are in line with those reported previously in Japan, Portugal, Italy, Argentina, the United States, and Uganda [20,21,22,23]. However, the FB_2_ levels produced by 27 strains of *A. niger* intended used in this study are significantly higher than that employed by Mogensen et al. (concentration: 2.9 μg/kg to 25 µg/kg on CYAS; 5.3 μg/kg to 36 µg/kg on RC; 0.46 μg/kg to 3.1 µg/kg on PDA) [19]. This may contribute to either different strains of *A. niger* or the media for toxin production.

Another interesting finding is that the positive rate and concentration of FB_2_ produced by *A. niger* in this experiment are strains function-dependent. Strains for saccharifying enzyme production produced higher level of FB_2_ than those for organic acid and tannase production on corn, rice and wheat bran. These might be due to the following two reasons. One is the difference in mycelium development among strains used for different functions. Strains intended for saccharifying enzyme production always grow faster than those of organic acid and tannase producers. Another reason is that there are big differences in mRNA expression level between high and low FB_2_ producers. High mRNA expression of eight toxigenic genes in six high FB_2_ producers of *A. niger* including three saccharifying enzyme producers (SN-03, SN-04 and SN-09) and three organic acid producers (OA-01, OA-03 and OA-05) were observed in comparison with those of lower FB_2_ producers. No differences in mRNA expression levels among six high FB_2_ producers were found. However, there is at least one gene with lower mRNA expression in strains of low FB_2_ producers, compared with those of high FB_2_ producers. Three genes *fum6*, *fum14* and *fum19* are the most common genes in relation to lower mRNA expression, which is in line with those reported by Palumbo [24]. In addition, it should be emphasized that we are still trying to knockout three key genes including *fum4*, *fum16* and *fum19* genes which might play the important role in FB_2_ production to elucidate the mechanism of FB_2_ biosynthesis by *A. niger*.

Besides this, it is worthy of pointing out that the strains of *A. niger* are with narrower fumonisin producing pattern and produce FB_2_ only, while the strains of *F. verticilliodes* are of broader fumonisin spectrum and could produce FB_1_, FB_2_ and FB_3_ simultaneously. This might be due to the reason that the strains of *A. niger* did not carry the *fum2* gene which encoded P450 oxygenase that was responsible for hydroxylation on C-10, a necessary step for FB_1_ and FB_3_ synthesis in *Fusarium* species such as *F. verticilliodes* and *F. proliferatum* [25,26,27]. 

Since the condition, especially the culture ingredients, for enzyme or organic acid production is different from one manufacturer to another, it is complicated to design an universal “real use” condition for co-production of enzyme or organic acid and FB_2_ at this moment. Meanwhile, most of strains used for the enzyme or organic acid production were in the liquid. Therefore, we can infer that the level of FB_2_ production under “real use” condition should be lower than those media used in this study. However, it is necessary to determine both enzyme (or organic acid) and FB_2_ simultaneously under “real use” condition in the near future in order to evaluate the safety of strains of *A. niger* accurately and scientifically. The Chinese food industry, like others in the world, has continued to experience increasing demand for food products. Our findings indicated that most of *A. niger* strains can produce FB_2_ on the natural media including corn, rice and wheat bran. This implies that invasion of corn, rice and wheat by *A. niger* in the field or during the storage may be contaminated with high level of FB_2_. On the other hand, cereal contamination with fumonisins will be contributed by both *Fusarium* and *A. niger*. All these data strongly emphasized the need for evaluation of the fumonisin producing ability for the strains of *A. niger* before they were used in Chinese food fermentation and in order to secure the absence of mycotoxin in the final industrial products. 

## 4. Materials and Methods

### 4.1. Chemicals and Reagents

Standards of FB_1_, FB_2_ and FB_3_ (Purity > 98%) were purchased from Romer Labs (IFA-Tulln, Tulln, Austria). Stock standard solutions were prepared in acetonitrile at the concentration of 50.4 µg/mL and diluted with acetonitrile and 0.2% formic acid (20:80, *v*/*v*). All organic solvents including methanol and acetonitrile used for sample extraction and UPLC-MS/MS analysis were of HPLC grade and purchased from Fisher Scientific (Fair Lawn, NJ, USA). Pure water was obtained from a Millipore Milli-Q system (Millipore, Bedford, MA, USA) with conductivity higher than 18.2 MΩ at 25 °C. 

### 4.2. Fungal Strains

Thirty strains of *A. niger* including 27 strains intended used in Chinese food industry such as saccharifying enzyme, tannase, galactosidase, as well as citric acid production, two strains used as control strains including one used for classification and another for antifungal activity determination, and one strain isolated from corn were purchased from China General Microbiological Culture Collection Center (CGMCC), Agricultural Culture Collection of China (ACCC), and American Type Culture Collection (ATCC), respectively. A total of 31 strains of *F. verticillioides* isolated from corn or wheat samples from Jiangsu, Anhui, Henan and Hebei and maintained on potato dextrose agar (PDA) were used for comparative study of fumonisin production between *A. niger* and *F. verticillioides*. 

### 4.3. Fumonisins Production

All strains of *A. niger* and *F. verticillioides* were inoculated on slants of both Czapek agar (CA) and PDA medium and incubated at 28 ± 1 °C for one week, respectively. Spore cultures suspension were made by adding 20 mL sterilized distilled water into the slant, scraping the hypha and spores and mixed thoroughly with inoculating hook. Flasks containing 100 g of polished rice, wheat bran or corn brought up to 20% (30% for wheat bran) relative moisture, respectively were autoclaved twice on successive days at 121 °C for 20 min and inoculated with 5 mL of one week old *A. niger* or *F. verticillioides* spore suspension or 5 mL sterilized distilled water used as control in two parallel, respectively and incubated in the dark at 28 ± 1 °C for 4 weeks. For the study of the time course, 10 grams of cultured material was collected on day 7, day 14, day 21, and day 28 and analyzed for fumonisins. All inoculated cultures were left standing for the first three days and thereafter shaken daily to reduce clumping. All the levels of fumonisins on the three media corn, rice and wheat bran used as control in this experiment were below limits of detection or not detected.

### 4.4. Fumonisin Extraction and Analysis

The extraction of fumonisins including FB_1_, FB_2_ and FB_3_ was modified based on the procedures published previously [28]. Briefly, 4 g of cultured material was homogenized with 20 mL of acetonitrile-water (50:50, *v*/*v*) and extracted for 60 min at 200 rpm on an orbital shaker (Eyela Inc., Tokyo, Japan) followed by sonicating for 1 h. The extracts were centrifuged at 10,000 rpm for 15 min (Beckman Coulter, Brea, CA, USA). An aliquot of 0.25 mL supernatant was diluted with 0.75 mL acetonitrile—0.2% formic acid in water (25:75, *v*/*v*), centrifuged again for 10 min at 10,000 rpm followed by filtrating through 0.45 µm PTFE filters (Jinteng, Tianjin, China). The filtrate was analyzed for FB_1_, FB_2_ and FB_3_ by UPLC-MS/MS (Waters, Milford, MA, USA).

### 4.5. UPLC Conditions

Detection and quantification of FB_1_, FB_2_ and FB_3_ were performed on a Micromass Quattro^®^ Premier XE LC-MS/MS system (Waters, Milford, MA, USA). The UPLC system consisted of Acquity ultra-performance liquid chromatography (Waters, Milford, MA, USA) with an Acquity UPLC BEH C_18_ column (100 mm × 2.1 mm i.d., 1.7 μm particle size, Waters, Milford, MA, USA) thermostated at 40 °C for separation. The mobile phase included acetonitrile (solvent A) and 0.1% formic acid in water (solvent B). A binary gradient at a flow rate of 0.35 mL/min was programmed starting at 40% A for 1 min, reaching 100% A in 4 min, and was maintained there for 2 min. Afterwards, B was linearly increased to 60% (*v*/*v*) within 0.1 min and maintained for 2.4 min. The injection volume was 10 µL and the sample temperature was maintained at 5 °C. The retention time of the three toxins were 1.38 min for FB_1_, 2.48 min for FB_2_ and 2.13 min for FB_3_, respectively.

### 4.6. MS/MS Conditions 

MS/MS was performed on an UltimaTM Micromass^®^-Quattro Premier XE triple-quadrupole mass spectrometer equipped with an electrospray ionization source (ESI, Waters, Milford, MA, USA). The mass spectrometer was operated in positive electrospray ionization (ESI^+^) mode for quantitation of the three fumonisins. The capillary voltage and cone voltage was set at 3.5 kV and 30 V, respectively. The source block temperature was 100 °C, and desolvation temperature was 350 °C. Nitrogen (purity = 99.9%) was used as a desolvation gas at a flow rate of 650 L/h. The collision gas pressure for nitrogen was 4.23 × 10^−3^ mbar and the dwell time was 0.2 ms using multiple reactions monitoring (MRM) mode. The parent ions (*m*/*z*) of FB_1_, FB_2_ and FB_3_ are 722.1, 706.1 and 706.1, respectively. The most intense product ion was employed as the quantifying ion, and the less intense signals were used as qualifying ion for confirmation of toxin identity. The quantitative daughter ions as well as collision energy in the parentheses (e*V*) are 334.1(32 e*V*) for FB_1_, 336.1 (35 e*V*) for FB_2_, 336.1 (35 e*V*) for FB_3_, respectively. The qualitative daughter ions (collision energy in the parentheses, e*V*) are 352 (32 e*V*) for FB_1_, 354.1 (35 e*V*) for FB_2_, 354.1 (35 e*V*) for FB_3_, respectively. Additionally, abundance ratios of MRM transitions as well as the chromatographic retention time enabled definite confirmation of the toxins presence. Data acquisition and evaluation was performed by Masslynx v4.1 (Waters, Milford, MA, USA). 

### 4.7. Preparation of Standard Solutions

Stock FB_1_, FB_2_ and FB_3_ standard solutions at the concentration of 500 µg/L were prepared by transferring 99.2 µL for each standard solution at the concentration of 50.4 mg/L into 10 mL volumetric flask, respectively, and stored at −18 °C. Working standard solutions were made by diluting the stock standard solutions with mobile phase. A matrix-matched calibration curve was used for the quantification of the three mycotoxins in different cultured materials in order to minimize matrix interference. Working standard solutions of FB_1_, FB_2_ and FB_3_ were added to fumonisin-free sample extract residue, reconstituted with 1 mL of acetonitrile—0.2% formic acid in water (20:80, *v*/*v*) and analyzed by UPLC-MS/MS. 

### 4.8. Method Validation

The mean recoveries in which matrix effect was compensated were determined from three parallel analysis of fumonisin-free corn, wheat bran and rice samples spiked with 0.6–400 μg/kg FB_1_, 0.6–400 μg/kg FB_2_ and 0.6–400 μg/kg FB_3_ standards were in the range between 77.78 ± 7.23%, 114.69 ± 9.81%, and 87.65 ± 10.81% for corn, 75.98 ± 8.21%, 96.29 ± 15.69% and 81.34 ± 11.32% for wheat bran, 120.34 ± 18.01%, 98.76 ± 5.84%, 79.41 ± 5.81% for rice, respectively. Method repeatability was determined by spiking toxin-free samples with the toxin standards at a concentration of 10 µg/kg for FB_1_, FB_2_, and FB_3_, respectively followed by extraction, purification and analysis by UPLC-MS/MS for six times a day. Reproducibility was determined by analyzing the spiked samples once a day on five successive days. The results indicated that the relative standard deviation ranged from 2.57% to 5.63% for intra-day and from 5.76% to 11.43% for inter-day. The limits of detection were 0.2 μg/kg for rice and corn, and 0.3 μg/kg for wheat bran for these three toxins, respectively.

## Figures and Tables

**Table 1:** FB_2_ production by *A. niger* intended use for food production on different media.

Stain No ^1^	Corn (μg/kg)				Rice (μg/kg)				Wheat Bran (μg/kg)			
	Day 7	Day 14	Day 21	Day 28	Day 7	Day 14	Day 21	Day 28	Day 7	Day 14	Day 21	Day 28
SN-01	12,802	70,488	33165	32,910	11,449	15,716	6699	10,982	970	2562	3915	3048
SN-02	6710	8166	6217.5	9442	3726	3116	2883	4462	3694	3442	4167.5	3490
SN-03	4192	4540	3855	4552	10,160	6648	5665	8876	718	860	828	866
SN-04	6304	8880	8482.5	8794	3518	4030	3640	5068	12,556	21,414	23,713	20,090
SN-05	1282	850	860	3776	1570	2364	1078	2720	158	198	268	266
SN-06	644	1970	3707.5	14,174	18,174	4252	34,994	54,284	150	240	175	186
SN-07	942	2046	1916	1288	1926	5734	3376	3232	6640	9756	10,354	13,826
SN-08	5264	11,978	7735	6592	4384	3036	3175	4892	10,558	18,148	12,355	15,360
SN-09	2534	11,854	14,105	16,042	320	554	278	482	31,994	18,050	19,248	25,028
SN-10	6	2	16	6	nd ^2^	4	12	10	110	30	36	62
SN-11	2336	3300	2694	4556	3968	4720	4448	3814	3052	8696	7232	6918
SN-12	2716	7878	8474	7534	4644	20,178	16,744	20,906	5558	7858	8790	8160
SN-13	458	1554	1416	1072	1786	568	324	410	1310	5304	3556	2922
OA-01	958	2874	4952	3978	1784	54	40	34	230	928	624	500
OA-02	608	220	78	98	250	246	230	4	7104	1212	180	48
OA-03	22	262	78	24,240	18	12	15	6	7018	13,370	14,193	8740
OA-04	332	7072	43,138	28,594	376	1990	9423	1996	34,250	22,326	13,735	23,498
OA-05	3376	1258	10,600	3268	368	1576	3426	4776	38,094	34,506	18 26	28,826
OA-06	nd	nd	123	nd	nd	nd	5	nd	nd	nd	nd	2
TA-01	5	10	3	10	nd	2	8	4	14	20	28	30
TA-02	nd	nd	nd	6	nd	nd	nd	nd	nd	nd	3	2
TA-03	nd	nd	4	2	nd	nd	nd	nd	nd	nd	nd	nd
TA-04	nd	4	nd	nd	4	nd	nd	nd	nd	nd	2	2
TA-05	nd	nd	nd	4	nd	8	nd	nd	nd	nd	5	4
TA-06	nd	nd	nd	6	nd	nd	nd	14	nd	nd	6	2
TA-07	nd	nd	nd	10	nd	nd	nd	nd	nd	nd	nd	nd
GA-01	2	4	4	2	nd	10	6	6	120	222	138	138
ACCC30557	256	1558	553	2194	1226	5764	1223	78	nd	16	8	4
ATCC16404	2	20	25	28	38	20	30	40	nd	nd	nd	2
SI-01	2050	5828	1335	13,342	11,188	22,906	20,873	23,700	1422	2382	8718	2174

**^1^SN 01 to SN-13**: saccharifying enzyme producer (*n* = 13); **OA-01 to OA-06**: organic acid producer (*n* = 6); **TA-01 to TA-07**: tannase producer (*n* = 7); **GA-01**: beta-galactosidase producer (*n* = 1); **SI-01** isolated from corn (*n* = 1). ^2^ nd: not detected.

**Table 2:** Mean levels of FB_2_ produced by 27 strains of *A. niger* intended used in Chinese food industry on corn, rice and wheat bran.

Substrate	Time Intervals (day)	FB_2_ Production (µg/kg)					
		Saccharifying Enzyme Producer (*n* = 13)		Organic Acid Producer (*n* = 6)		Tannase Producer (*n* = 7)	
		No. of Positive (%)	Mean (Range) (μg/kg)	No. of Positive (%)	Mean (Range) (μg/kg)	No. of Positive (%)	Mean (Range) (μg/kg)
Corn	7	13 (100)	3553 (6–12802)	5 (83)	1059 (22–3376)	1 (14)	5
	14	13 (100)	10,270 (2–70,488)	5 (83)	2337 (220–7258)	2 (29)	7 (4–10)
	21	13 (100)	7126 (16–33,165)	6 (100)	9828 (78–43,138)	2 (29)	3 (3–4)
	28	13 (100)	8518 (6–32,910)	5 (83)	12,036 (98–28,594)	5 (71)	6 (2–10)
Rice	7	13 (100)	5469 (320–18,174)	5 (83)	559 (18–1784)	1 (14)	4
	14	13 (100)	5455 (4–20178)	5 (83)	776 (12–1990)	2 (29)	5 (2–8)
	21	13 (100)	6409 (12–34,994)	6 (100)	2190 (5–9423)	1 (14)	8
	28	13 (100)	9241 (10–54,284)	5 (83)	1363 (4–4776)	1 (14)	9 (4–14)
Wheat bran	7	13 (100)	5959 (110–31,994)	5 (83)	17,339 (230–38,094)	2 (29)	14
	14	13 (100)	7428 (30–21,414)	5 (83)	14,468 (928–34,506)	1 (14)	20
	21	13 (100)	7280 (36–23,713)	5 (83)	9491 (180–18,726)	4 (57)	9 (2–28)
	28	13 (100)	7709 (62–25,028)	6 (100)	10269 (2–28,826)	5 (71)	8 (2–30)
